# Endosphere microbial communities and plant nutrient acquisition toward sustainable agriculture

**DOI:** 10.1042/ETLS20230069

**Published:** 2023-11-17

**Authors:** Olubukola Oluranti Babalola, Afeez Adesina Adedayo

**Affiliations:** Food Security and Safety Focus Area, Faculty of Natural and Agricultural Sciences, North-West University, Private Mail Bag 2046, Mmabatho, South Africa

**Keywords:** microbial diversity, PGPR, plant tissue, root adherence, seed, yield enhancement

## Abstract

Endophytic microbial communities have essential information for scientists based on their biological contribution to agricultural practices. In the external plant environment, biotic and abiotic factors affect microbial populations before getting into plant tissues. Endophytes are involved in mutualistic and antagonistic activities with the host plant. Microbial communities inhabiting the internal tissues of plant roots depend on their ability to live and contend with other plant microflora. The advantageous ones contribute to soil health and plant growth either directly or indirectly. The microbial communities move via soil–root environment into the endosphere of plants promoting plant growth features like antibiosis, induced systemic resistance, phytohormone synthesis, and bioremediation. Therefore, the existence of these microorganisms contributes to plant genomes, nutrient availability in the soil, the presence of pathogens, and abiotic factors. This review aims at how endophytic microorganisms have displayed great interest in contributing to abundant crop production and phytopathogen inhibition.

## Introduction

In plant tissues, the region where endophytes dwell is known as the endosphere [1]. Studies on endophytic microbial communities in exploring their benefits to promote the cultivation of crops are favorable characteristics in the farming system. Much research has revealed the application of endophytic microbiotas in the food insecurity sector [2–4]. Endophytic bacteria initiate a mutualistic and antagonistic relationship with beneficial plants known as plant growth-promoting endophytes (PGPEs) [5]. The PGPEs improve plant growth by making available major nutrients required for plant growth in the soil, fixing nitrogen, phytohormone production, and chelating compound synthesis that activate the control of plant pathogens due to plant response [6]. Some endophytic bacteria like Bacillus, Brevibacillus, Acinetobacter, Agrobacterium, Herbaspirillum, Burkholderia, Azospirillum, and Pseudomonas have been used as bioinoculants [7]. As a result of environmental challenges caused by the application of chemical fertilizers, the application of endophytic bacterial inoculants as a sole alternative represents an effective process of improving a stable environment [8]. Omomowo and Babalola [9] reported how *Stenotrophomonas geniculata* NWUBe21 and *Pseudomonas carnis* NWUBe30 is an endophytic bacteria reported to express specific functional characteristics to promote metabolic activities and support the plant adaptation to different environmental factors of the habitat it is inhabiting. Other species of endophytic bacteria are facultative anaerobes, rod-like gram negative, and spore forming bacteria and are commonly isolated in plant–soil zone.

Endophytic microbes are known as a member of rhizosphere microbial communities that infiltrate plant root tissues through seed inoculation by vertical methods or from rhizosphere soil to plant tissue via horizontal means [10]. The infiltration of endophytic microorganisms is alleviated by the production of substances containing signal molecules to obtain nutrients in the soil–root ecosystem [11]. The plant root system ascertains exudates that comprise biomolecules like sugar, carbohydrates, vitamins, nucleotides, volatile materials, enzymes, amino acids, flavonoids, phenolics, and fatty acids as carbon sources for the microbial communities. Endophytic microbial communities assist in exchanging materials produced in response to reciprocal actions between exudates produced from plant roots and microorganisms. This interaction promotes plant growth and improves the health status of plants [10]. Endophytes were reported to deduce the effect of abiotic stresses, including high temperature, drought effect, nutritional deficiency, and biotic stress like the invasion of phytopathogens [10].

Endophytic microbes, including bacteria, fungi, archaea, and actinomycetes, are likewise known as bionanomaterials. This is because the microbes can assimilate metallic ions and change their state to compound from elements. Changing their states can be intracellular or extracellular; microbes classify the synthesized nanomaterials. However, microorganisms do produce secondary metabolites for the biosynthesis of nanoparticles from metals like silver, gold, platinum, palladium, selenium, magnetite, etc. [12].

Therefore, the assimilation of nutrients by plant-related microbial communities capacitates them to utilize good values on the host plant. This review shows the potential of endophytic bacteria and fungi as bioinoculants improving the growth of plants and contributing to sustainable agriculture.

## Endophytic microorganisms in plant

**Figure 1.**
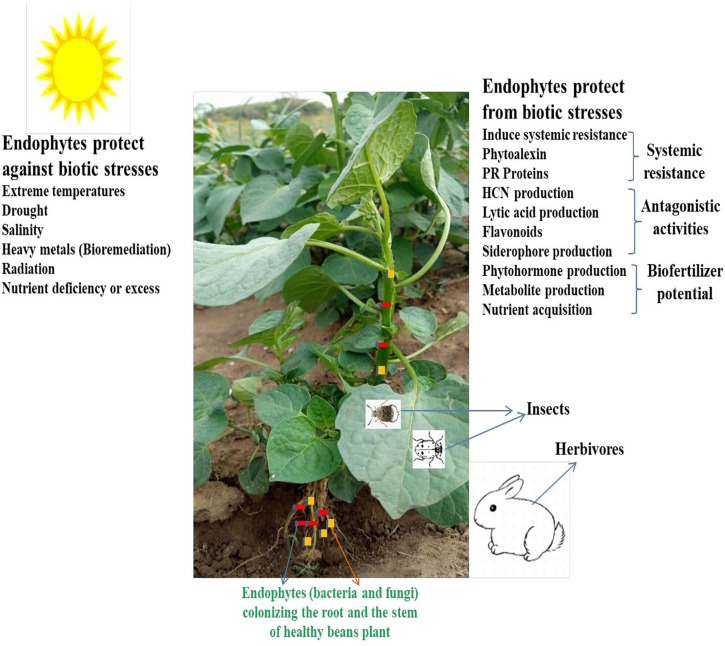
The potential of endophytic microbes in the leguminous plants tissues The potential of endophytic microbes in the roots and stems of beans plants and shedding of microbes from the root tissues into the rhizosphere soil.

Various plant organs, including roots, stems, branches, and leaves, shelter different microbes that involve intimate association with the host plant (Figure 1). Endophytes are related to some plants like wheat, maize, soybean, sorghum, etc., of which the culturable microbes inhabit the endosphere of the plant [5]. The essential bacteria influencing the plant endosphere are gram-positive and negative, but endophytic arbuscular mycorrhiza fungi are found in the rhizosphere, forming intimate associations with plant roots [6]. Endophytic microbial (bacteria and fungi) diversity [13], functional diversity [14], and functional genes [15] dwelling in the endosphere of different organs of plants has been characterized. According to Fadiji et al. [13], some microbial genera were reported to be abundant in maize fertilization sites, including *Flavobacterium*, *Paenibacillus*, *Pseudomonas*, *Bacillus*, *Pedobacter*, *Chryseobacterium*, *Corynebacterium*, *Acinetobacter*, and *Brevibacillus* in the maize root rhizosphere (Table 1).

**Table 1.** **Endophytic microorganisms in plant** **endosphere and their functions**

Endophytic microbes (bacteria and fungi)	Crops	Functional activities	References
*Stenotrophomonas, Brevundimonas, Staphylococcus, Paenibacillus, Bacillus, Myroides, Lynsinibacillus, Pseudomonas, Mammalicoccus, Micrococcus,* and *Ignatzscineria*	Cowpea (*Vigna unguiculata*)	They involve in plant growth promotion	[3]
*Stenotrophomonas maltophilia* JVB5	Sunflower (*Helianthus annuus*)	Reveals predicted multifunctional genes that improve the sustainability of sunflower plants	[1]
*Paraburkholderia phytofirmans*	–	These endophytes are bioinoculants and prevent phytopathogen invasions	[2]
*Bacillus, Pseudomonas*, and *Stenotrophomonas*	Sunflower (*Helianthus annuus*)	These PGPR produce hydrogen cyanide, indole-3-acetic acid (IAA), ammonia, siderophore, exopolysaccharide, and solubilize phosphate	[4]
Bacteria (*Colletotrichum* sp., *Bacillus cereus*, *Penicillium citrinum*, *Pseudomonas veronii*) and fungi (*Aspergillus fumigatus*, and *Saccharomonospora* sp.)	–	The microorganisms produce secondary metabolites which give them the potential to produce nanoparticles	[12]
*Acidovorax, Flavobacterium, Hydrogenophaga,* and *Burkholderia-Paraburkhoderia*	Sunflower (*Helianthus annuus*)	The bacteria reveal their bioinoculant potential on sunflower plants	[7]
*Bacillus cereus* T4S	Sunflower (*Helianthus annuus*)	The PGPR produces genes that contribute to the growth of plants	[16]
Endophytic archaea genra (*Haloferax, Candidatus Nitrososphaera, Methanobacterium, and Thermoplasma*), endophytic fungi genera (*Filobasidiella, Ustilago, Tilletia, Metarhizium, Sordaria, Coprinopsis, Sclerotinia, Gibberella, Phaeosphaeria, Podospora, Ajellomyces, Aspergillus, Schizosaccharomyces, Talaromyces, Phaeosphaeria,* and *Leptosphaeria*)	Maize (*Zea mays*)	Endophytic archaea and fungi diversity were reported to contribute to maize growth on a sample of organic fertilizer site	[17]
Endophytic bacteria genera (*Bacillus, Chitinophaga, Pedobacter, Chryseobacterium, Flavobacterium, Dyadobacter, Paenibacillus, Pseudomonas, Corynebacterium, Brevibacillus*, and *Acinetobacter*)	Maize (*Zea mays*)	The endophytic bacteria were abundant in fertilization sites promoting the growth of plants	[13]
*Bradyrhizobium sp* SUTNa-2, *Enterobacter cloacae* RCA25, *Herbaspirillum huttiense* RCA24, *Pseudomonas granadensis* T6; *Rhizobium larrymoorei* E2	Rice (*Oryzae sativa*)	Promote the growth of rice plants and pesticide tolerance	[11]

### Acquisition of nutrients by plants

Plants acquire nutrients from the soil and the atmosphere. The essential nutrients for plant growth include macronutrients such as nitrogen (N), phosphorus (P), potassium (K), calcium (Ca), magnesium (Mg), and sulfur (S), and micronutrients such as iron (Fe), manganese (Mn), zinc (Zn), copper (Cu), boron (B), molybdenum (Mo), and chlorine (Cl) [18]. Plants take up these nutrients in different ways. Macronutrients are typically taken up as ions from the soil solution by the plant roots through active or passive transport mechanisms. Active transport involves using energy to move ions against a concentration gradient, while passive transport involves the movement of ions down a concentration gradient [19]. Micronutrients are taken up similarly, but they are often more tightly bound to soil particles and may require the help of specialized transporters or chelators to be taken up by the plant [20].

Plants also acquire nutrients through symbiotic relationships with microorganisms in the soil, such as mycorrhizal fungi and nitrogen-fixing bacteria [21]. Mycorrhizal fungi form a mutualistic association with plant roots, providing the plant with increased nutrient uptake, particularly phosphorus, in exchange for carbohydrates from the plant [22]. Nitrogen-fixing bacteria, such as *Rhizobium* spp., form symbiotic associations with legumes, converting atmospheric nitrogen into a form usable by the plant [23]. Plants can also acquire nutrients from the atmosphere through their leaves. For example, atmospheric carbon dioxide is converted into organic carbon through photosynthesis, providing the building blocks for plant growth. Additionally, some plants can absorb nutrients, such as nitrogen and sulfur, through their leaves in the form of gases, such as ammonia and sulfur dioxide [24]. This means the acquisition of nutrients by plants is a complex process that involves various mechanisms, including uptake from the soil, symbiotic relationships with microorganisms, and uptake from the atmosphere. Understanding these mechanisms is essential for optimizing plant productivity and developing sustainable agriculture practices.

### Endophytic microorganisms involved in the acquisition of nutrients by plants

Endophytes are microorganisms that live inside plant tissues without causing any harm to the host plant. However, some endophytes play a crucial role in nutrient acquisition by the host plant. Here are a few examples:

Mycorrhizal fungi form a mutualistic association with the roots of most plants. The fungi colonize the root system and form a network of hyphae that extend into the soil, increasing the surface area available for nutrient uptake [25]. The fungi absorb water and mineral nutrients from the soil and transfer them to the host plant. In return, the plant provides the fungi with carbon compounds.

Examples of PGPEs include bacteria like *Bacillus*, *Pseudomonas*, and *Rhizobium*. The bacteria infect the root system of the plant and form nodules, where they fix atmospheric nitrogen into a form that the plant can use [26]. In return, the plant provides the bacteria with carbon compounds. Rhizobia are a group of soil bacteria capable of forming a symbiotic relationship with certain plants, particularly legumes, by colonizing the roots and forming specialized structures called nodules [27]. Within these nodules, the bacteria convert atmospheric nitrogen into a form the plant can use in exchange for carbohydrates from the plant. This symbiotic relationship is known as nitrogen fixation and is vital for soil fertility and plant growth. Some examples of Rhizobia are *Rhizobium leguminosarum* which forms symbiotic relationships with legumes such as peas, beans, and clovers [28]; *Bradyrhizobium japonicum*, commonly found in soybean nodules and is essential for nitrogen fixation in soybean crops [29]. *Sinorhizobium meliloti* forms symbiotic relationships with alfalfa and other legumes and is used in agriculture to improve soil fertility [30], and *Mesorhizobium loti* is known to form symbiotic relationships with Lotus species [31].

Largely, rhizobia play a crucial role in improving soil fertility and the growth of legume crops and are an important contributor to sustainable agriculture. Some endophytic bacteria live inside the tissues of plants and help in nutrient acquisition. For example, *Bacillus subtilis* can produce siderophores that chelate iron, making it available to the plant [32]. Other endophytic bacteria can produce plant growth-promoting hormones, such as indole acetic acid (IAA), stimulating root growth and nutrient uptake.

Endophytic fungi can also contribute to nutrient acquisition in plants. For example, some endophytic fungi can solubilize phosphorus, making it available to the host plant [33]. Other fungi can produce enzymes that break down complex organic compounds, releasing nutrients that the plant can take up. Examples of PGPEs include fungi like *Trichoderma*, *Penicillium*, *Aspergillus*, etc.

PGPEs can be found naturally in the soil or applied to plant roots through inoculation methods such as seed treatment, soil drenching, or foliar spraying. The modification of endophytes is a novel method of nutrient acquisition by plants to yield abundant production of crops. Various microbial inoculation methods including introduction into plant tissues and soil, introduction into the plants’ seeds, and atomization of various tissues of plants were presented by Adeleke *et al.* [2].

## Factors influencing microbial communities diversity in plant

Plant microbial community diversity refers to the variety and abundance of microorganisms, such as bacteria, fungi, and viruses, that interact with plant roots and leaves in the soil, rhizosphere, and phyllosphere [34,35]. Microbial communities survive in the endosphere and depend on their potential to inhabit and produce certain metabolites [8]. Microbial diversity ranges in various crops, i.e. the diversity of microbes in cowpea is different from maize plants. Nevertheless, phylogenetic similarities in plants may possess different endophytic microorganisms and therefore attached to the nutrient acquisition of the plants [14].

The plant microbiome plays an essential role in plant growth, health, and stress tolerance by providing nutrients, protecting against pathogens, and regulating plant physiology. Studies have shown that a diverse and stable microbial community can enhance plant growth, increase nutrient uptake, and reduce susceptibility to diseases and abiotic stresses.

The diversity of endophytic microbiota is determined by some factors like soil, the nature of the endophytes, plants, and environmental factors [6] and their genomics assessment (Table 2). Factors that affect plant microbial community diversity include plant species, soil type, climate, management practices, and biotic and abiotic stressors. For example, different plant species may select specific microbial communities based on their root exudates and other chemical signals [40]. At the same time, management practices such as tillage and fertilizer application can alter the soil microbiome.

**Table 2.** Genomic data sequence assessment of endophytic microorganisms

Endophytic microbes	Plant host	Activities	References
—	Sunflower endosphere	Investigate amplicon metagenome sequencing employing Illumina MiSeq (Bioproject accession number: PRJNA673781 and PRJNA673791)	[36]
*Bacillus cereus* T4S	Sunflower root	Conduct whole genome sequence of root microbe (BioProject sample https://www.ncbi.nlm.nih.gov/bioproject/PRJNA706601)	[37]
*Stenotrophomonas indicatrix* BOVIS40 and *Stenotrophomonas maltophilia* JVB5	Sunflower roots	Genomic sequencing of endophytic microbes and the genes produced for plant growth promotion (BioProject accession number PRJNA706595 and PRJNA706608)	[38]
*Bacillus* spp., *Chitinophaga*, *Flavobacterium*, *Chryseobacterium*, *Paenibacillus*, *Pedobacter*, and *Alphaproteobacteria*	Maize roots	Shotgun metagenomic sequencing of the plants’ roots (Bioproject number PRJNA607664)	[39]

### Antibiosis

The features of metabolites obtained from endophytes reveal the antibiotic activities of endophytic microorganisms that are manufacturers of specific bioactive materials that have been observed for several years. These materials showed the level of minimum inhibitory concentration (MIC) i.e. the minimum rate at which they can be applied to reduce or inhibit the invasion of phytopathogens on the crops. Endophytes have been reported on medicinal plants to produce an extract of ethyl acetate that shows absolute inhibition and prevention of diseases on crops [41]. The potential of the bioactive metabolic material produced by the endophytic microorganisms (*Bacillus aerophilus*, *Pseudomonas entomophila*, and *Penicillium chrysogenum*), especially in medicinal plants like *Moringa oleifera* and *Aloe vera* acts against various phytopathogens like *Bacillus cereus*, *Candida albicans*, *Klebsiella pneumonia*, *Staphylococcus aureus*, *Escherichia coli*, *Pseudomonas aeruginosa*, etc. [42].

### Endophytic organisms improve soil health and plant growth

Endophytes are cosmopolitan and found in every healthy plant. They induce growth in plants and stimulate plant physiology, and the physiochemical characteristics of the soil through direct and indirect mechanisms [43,44]. The following activities were carried out by endophytes in plants; solubilization of phosphorus, production of exopolysaccharides and exoenzymes, and antagonistic activities which further contribute to the fertility and health status of agricultural soil [45]. The exoenzymes secreted possess the potential of solubilizing major nutrients required by plants from the insoluble to the soluble form. They also contain organic acids that reduce the acidity of the soil. The reduction in the soil acidity, however, reduces the invasion of phytopathogens on the plants and improves the development of plants [46]. Some endophytic microbes manufacture soil enzymes like urease, alkaline phosphate, and invertase that proportionally control soil organic carbon (SOC), microbial population, and the amount of nitrogen present in the soil [47]. The degradation of plant wastes that are complex sugar like cellulose, lignin, hemicellulose, pectin, oligosaccharides, proteins, and lipids in the soil was also done by endophytes thereby producing simpler molecules [48]. The molecules produced contributed to the health status of the soil by adding nutrients, returning beneficial microbes to the soil, and promoting the quality of the soil.

### Induced systemic resistance

Induced systemic resistance (ISR) is a plant's enhanced ability to defend itself against pathogens, insects, and other stress factors following initial exposure to specific chemical or biological agents. ISR is a complex mechanism that activates multiple defense pathways within the plant [49]. These pathways can involve changes in the expression of genes related to defense responses, the production of phytohormones such as salicylic acid, jasmonic acid, and ethylene, and the synthesis of various secondary metabolites with antimicrobial properties [50]. ISR can be induced by various agents, including beneficial microbes such as rhizobacteria and mycorrhizal fungi and certain chemical compounds, such as plant growth regulators, signaling molecules, and elicitors.

ISR is considered an attractive alternative to conventional chemical-based disease control methods because it is environmentally friendly, sustainable, and has no negative impact on human health or the ecosystem [51]. ISR-based approaches are increasingly being studied and developed for use in agriculture, horticulture, and forestry to enhance crop productivity and reduce the use of synthetic pesticides. Endophytic microorganisms elicit ISR also attached to the genes promotion that is revealed in pathogenesis [52]. The induction of systemic resistance inhibits the invasion of pathogens on plants. They are also known to alleviate abiotic stress through nanomaterials produced by the endophytes [53]. Specific endophytes like *Fusarium solani* inhabiting tomato roots induce ISR against foliar pathogen causative agents called *Septoria lycopersici* thereby activating PR genes in tomato roots [53].

### Phytohormone synthesis

The synthesis of phytohormone in plants contributes to signaling in plant–endophyte associations. The associations are of two types including plant growth-promoting characters like the production of IAA that controls the plant's growth and development via tissue differentiation, cell elongation, and division, phototropism, apical dominance [54], and 1-aminocyclopropane-1-carboxylate (ACC) deaminase that promote the growth of plants by reducing the ethylene level in plants [55]. Endophytic organisms also helped plants produce siderophores that helped scavenge minor elements (Fe, Co, Ni, Mn, Mo) required by plants [56] and cytokinin that contribute to the division of cells and improve the proliferation of leaf cells [57]. Endophytic microbes involve in host-specific adaptations and mutualistic association with plants [58]. This association can make plants tolerate the microbes and on the other way round, the microbes carry out metabolism processes in plants.

### Bioremediation

Endophytes are environmentally friendly microbes that improve the population of plant growth and prevent the invasion of diseases [52]. Endophytic microorganisms are involved in significant processes in the field of medicine, agriculture, and various industries. The roles of the endophytes promote plant health and the production of crops. These microorganisms are much important due to their potential for plant existence in stressed or harsh environmental conditions. The microbes also have the potential to scavenge the exudate produced from plant roots while interacting with the plants [59]. Endophytes have been employed on an industrial base to produce enzymes and antibiotics for the process of detoxification of metals in plants [52]. Biotransformation of chemicals reduces bioremediation and degradation of the polluted environment [60]. Endophytic microbes can fix nitrogen by converting atmospheric nitrogen into ammonium ions (NH_4_^+^) that are absorbable by plants, hence they are diazotrophic [61].

The process of detoxifying heavy metals from effluent produced by industries employing microorganisms is known as bioremediation technology. Bioremediation of metals by endophytic microbes needs to be known with the toleration of metal features [62,63]. The microbes promote plant growth in heavy metal and salt-stressed environments. The application of endophytic microorganisms in stressed agricultural environments has gained much popularity. The use of endophytes to combat environmental stresses is regarded as the most useful method for bioremediation because it is safe and generally acceptable [64]. Moreover, some microbes have revealed their potential to improve the storage of metals and further promote gene expression [65]. The regulation of ethylene in plants by the endophytes can affect heavy metal toleration by changing the impact of stresses [66]. The diversity of endophytic microbiomes and their functions (protein metabolism, stress response, membrane transport, regulation, cell signaling, carbohydrates, etc.) have been obtained via metagenomic sequencing [14].

## Biotic and abiotic stress affecting plant tissues

### Biotic stress

Various parasitic microbes known as pathogens affect plants by feeding on them as a result of biotic stress. These microbes belong to the following bacteria genera; *Diptera*, *Hemiptera*, *Orthoptera*, etc [67]. Fungi species cannot be left out among the organisms causing diseases in plants. They were known for their potential in inhibiting plant growth by either destroying the plant cell via secretion of toxigenic substances or feeding on the plant tissues. Common examples of these fungi are *Fusarium*, *Aspergillus*, and *Penicillium* [68]. Viruses have likewise played a major role in the destruction of plant organs. They cause chlorosis of leaves, soft rot on tomato fruits [69], powdery mildew on tomato leaves, stems, and branches [70], and diminished growth on potatoes [71] and cucumber plants [72]. Moreover, nematodes are other soil microbiomes that feed on plant organs and also invent wounds on the parts [73]. Other biotic agents that destroy 40% of plants globally are pests reported to ruin plants in agricultural systems.

Plants known to contain various endophytes in their organs produce certain defense mechanisms to combat pathogens’ invasion [74]. These defense mechanisms are carried out by certain metabolites produced by endophytic organisms. So that when pathogens invade, natural immunity is triggered against the pathogens thereby disallowing their invasion of plants. A plant tissue, a cuticle found on the outermost layer of plants skin, prevents liquid material or gases from entering the plants to protect the plants from spoilage organisms and pest attacks. However, plants have shown their potential of secreting metabolites (phenolics, alkaloids, amines, glucosides, quinines, etc.) to protect them from pathogens and herbivores [75]. The mentioned metabolites also reduce the level of disease occurrence in plants.

### Abiotic stress

Abiotic stress refers to any environmental stressor that affects plants’ growth, development, and productivity, but is not caused by living organisms. Abiotic stresses include extreme temperatures, drought, salinity, heavy metals, radiation, and nutrient deficiency or excess. These stressors can lead to changes in plant physiology, metabolism, and gene expression, ultimately resulting in reduced plant growth, yield, and quality [76]. For example, drought stress can cause a decrease in water availability, leading to reduced photosynthesis, stomatal closure, and oxidative damage to cells. Salinity stress can lead to ion toxicity and osmotic stress, decreasing water uptake, nutrient imbalance, and cellular damage.

Plants have evolved various mechanisms to cope with abiotic stress, including gene expression changes, ion transport and water uptake regulation, and the synthesis of various osmoprotectants and antioxidants [77]. Plant breeders and biotechnologists are also developing new strategies to enhance plant stress tolerance through genetic engineering, breeding, and biotechnology approaches.

## Future prospect

Various studies have been investigated on endophytic microbes but some challenges observed during the study cannot be overlooked. Antimicrobial materials produced by the endophytes revealed antifungal potential but cannot be utilized as fungicides or medical drugs. In culture medium, the application of the antimicrobials is not enough for commercial purposes. Antimicrobials do produce toxic substances that are deadly to other beneficial organisms. The materials are not specific because they can be toxic to humans too. Endophytes produce antimicrobials that assist in the biological synthesis of antimicrobials and control the existence of phytopathogens but these antimicrobials are unknown.

However, the substantial intention of investigating endophytic microbes is to obtain novel antimicrobials that are biologically active and have no negative effects on plants, animals, and humans. To improve the antimicrobial potential or reduce the consequences that can occur as a result of metabolites produced to promote the effectiveness of the microorganisms. Also, the investigation reveals the regulatory genes in producing antimicrobial materials and applying genetic technology to promote the acquisition of antibiotics. As a result of various antimicrobials obtained from endophytes, there is a chance of getting dependable and pleasant antibiotics that can be used clinically in the future.

## Conclusion

PGPEs are microorganisms, such as bacteria or fungi that live within plant tissues without causing any harm to the host plant. Instead, these endophytes can positively impact plants’ growth, development, and health by providing them with beneficial substances such as hormones, nutrients, or enzymes and protecting them against pests, diseases, and pesticides, thus promoting sustainable agriculture. Understanding the plant microbiome and its interactions with the environment is critical for developing sustainable agriculture practices, including biological control of pests and diseases, soil health management, and producing microbial-based fertilizers and biostimulants. In addition, advances in high-throughput sequencing and other omics technologies provide new opportunities to study the plant microbiome and its potential applications for improving plant productivity and environmental sustainability.

Endophytic microorganisms (bacteria, fungi, archaea) have revealed their ability to contribute to plant growth, antagonize disease invasion of plants, prove biopesticide, and bioherbicide, and stimulate tolerant characters to abiotic stresses. Managing abiotic stress is critical for ensuring sustainable agricultural production, environmental conservation, and addressing the challenges of climate change and land degradation. Using PGPEs represents a promising strategy for improving plant growth and health while reducing the environmental impact of agriculture.

## Summary

- Endophytes are involved in mutualistic and antagonistic activities with the host plant.
- Their ability to live and contend with other plant microflora.
- They contribute to soil health and plant growth either directly or indirectly.
- They can move through the soil–root environment into the endosphere of plants promoting plant growth features like antibiosis, induced systemic resistance, phytohormone synthesis, and bioremediation.

## Abbreviations

IAA: indole acetic acid
ISR: induced systemic resistance
PGPEs: plant growth-promoting endophytes
